# *APOE* and Alzheimer’s Disease: Neuroimaging of Metabolic and Cerebrovascular Dysfunction

**DOI:** 10.3389/fnagi.2018.00180

**Published:** 2018-06-14

**Authors:** Jason A. Brandon, Brandon C. Farmer, Holden C. Williams, Lance A. Johnson

**Affiliations:** Department of Physiology, University of Kentucky, Lexington, KY, United States

**Keywords:** ApoE, apolipoprotein E, cerebral, metabolism, brain, imaging, neurodegeneration, Alzheimer’s Disease (AD)

## Abstract

Apolipoprotein E4 (ApoE4) is the strongest genetic risk factor for late onset Alzheimer’s Disease (AD), and is associated with impairments in cerebral metabolism and cerebrovascular function. A substantial body of literature now points to E4 as a driver of multiple impairments seen in AD, including blunted brain insulin signaling, mismanagement of brain cholesterol and fatty acids, reductions in blood brain barrier (BBB) integrity, and decreased cerebral glucose uptake. Various neuroimaging techniques, in particular positron emission topography (PET) and magnetic resonance imaging (MRI), have been instrumental in characterizing these metabolic and vascular deficits associated with this important AD risk factor. In the current mini-review article, we summarize the known effects of *APOE* on cerebral metabolism and cerebrovascular function, with a special emphasis on recent findings via neuroimaging approaches.

## Introduction

Apolipoprotein E (ApoE) plays an critical role in the metabolism of lipoproteins and redistribution of cholesterol, and has long been studied in relation to atherosclerosis and cardiovascular disease (Mahley and Rall, 2000; Eichner et al., 2002; Pendse et al., 2009). In the periphery, apoE is primarily produced by the liver, but is also expressed by a number of other tissues (Driscoll and Getz, 1984; Zechner et al., 1991). In the brain, apoE is primarily produced by astrocytes, and it plays a critical role in neuronal maintenance and repair (Xu et al., 1996, 2006; Mahley and Rall, 2000). In humans, there are three major isoforms of apoE: E2, E3 and E4 (Mahley and Rall, 2000). E3 is the major isoform expressed in humans, and the effects of E2 and E4 are typically compared to those of E3 to determine relative risk (Phillips, 2014). Importantly, *APOE* is the strongest genetic risk factor for late onset Alzheimer’s Disease (AD), with E4 conferring between a 3- (heterozygous) to 15-fold (homozygous) increase in risk of AD (Farrer et al., 1997; Raber et al., 2004). Conversely, E2 is associated with increased longevity and a decreased risk of AD (Farrer et al., 1997; Garatachea et al., 2014).

Normal synaptic function requires a multitude of energy-intensive processes, and a complex and intricately linked interplay between neurons and supporting glia is necessary to maintain efficient energy metabolism (Belanger et al., 2011). Metabolic dysfunction, as in the case of insulin resistance (IR) and type 2 diabetes, increases the risk of dementia and shares several pathological characteristics with AD, such as inflammation, increases in oxidative stress and vascular dysfunction (Craft, 2009; Walker and Harrison, 2015). Metabolic disorders also increase in incidence with age (Narayan et al., 2006) and a rapidly ageing demographic means the number of individuals suffering from both metabolic disorders and AD is expanding precipitously.

**Figure 1:**
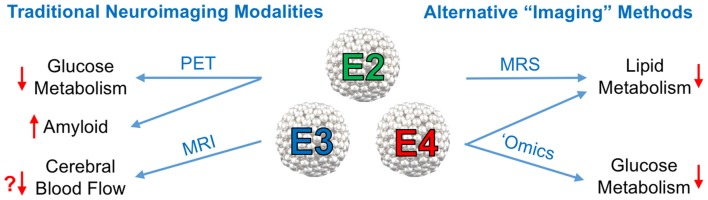
Neuroimaging approaches to study the effects of the Apolipoprotein E (ApoE) isoforms on cerebral metabolism. Left: traditional neuroimaging techniques, in particular positron emission topography (PET) and magnetic resonance imaging (MRI), have been invaluable in characterizing the metabolic and cerebrovascular deficits associated with APOE4, the strongest genetic risk factor for late onset Alzheimer’s disease (AD). ^18^F-Fluorodeoxyglucose positron emission topography (FDG-PET) imaging has consistently shown a pattern of brain glucose hypometabolism in individuals with E4, while amyloid PET imaging shows increased amyloid deposition. Some, but not all, blood oxygen level dependent (BOLD) functional magnetic resonance imaging (fMRI) studies in E4 individuals have shown decreased cerebral blood flow (CBF). Right: other alternative, or non-imaging methods of visualizing vascular and metabolic changes may prove instrumental in elucidating apoE isoform-specific effects on AD risk and progression. Magnetic resonance spectroscopy (MRS), metabolomics and lipidomics studies have implicated E4 in multiple pathways of lipid and glucose metabolism (effects of E4 denoted by red arrows).

It is now well established that E4 is associated with various impairments in CNS metabolism, most notably decreased cerebral glucose uptake. A substantial body of literature now suggests that E4 carriage can in itself be viewed as a form of cerebral metabolic dysfunction. In the current mini-review article, we summarize important recent findings related to apoE’s role in modulating cerebral metabolism and cerebrovascular function, with a special emphasis on neuroimaging approaches (see Figure 1).

## *APOE*, Cerebral Glucose Metabolism, and Peripheral Glucose Regulation

^18^F-Fluorodeoxyglucose positron emission topography (FDG-PET) is commonly used to measure cerebral glucose metabolism. A reduction in cerebral metabolic rate of glucose (CMRglc), as measured by FDG-PET, is now considered one of the hallmarks of AD (Small et al., 2000). FDG-PET is able to differentiate AD from other types of dementia with a high degree of specificity due to specific regional patterns (Laforce and Rabinovici, 2011). Clinical AD symptoms essentially never occur without glucose hypometabolism, and the extent of the metabolic changes are strongly correlated with the severity of clinical symptoms (Grady et al., 1986; Haxby et al., 1990; Blass, 2002). Furthermore, recent evidence suggests that these alterations in glucose metabolism occur very early in the neurodegenerative process (Small et al., 1995; Reiman et al., 1996; de Leon et al., 2001; Mosconi et al., 2008).

Interestingly, a pattern of brain glucose hypometabolism regionally similar to that observed in AD has been described in individuals with E4 in a number of studies over the past two decades (reviewed in more detail here, Wolf et al., 2013). This pattern of decreased cerebral glucose metabolism is observed even in non-demented, cognitively normal E4 carriers (Small et al., 1995, 2000; Reiman et al., 1996, 2001) thereby lending support to this being an inherent biological feature of E4, rather than simply a byproduct of dementia (Reiman et al., 2004). Importantly, these metabolic deficits are present decades in advance of AD onset in E4 individuals—reductions in cerebral glucose utilization are observed in normal E4 individuals as young as their 20–30s (Reiman et al., 2004). This pattern of cerebral glucose hypometabolism in young E4 carriers is considered the earliest brain abnormality described to date in living individuals at risk for AD (Mosconi et al., 2008). Below, we summarize some more recent findings that may shed light on this well-established E4-associated phenomenon, including studies that look beyond FDG-PET imaging.

A recent study by Nielsen et al. (2017) examined how peripheral apoE levels affect cognition, gray matter volume (GMV) and cerebral glucose metabolism in an isoform-dependent manner. They showed that females not only have higher plasma levels of total apoE and apoE4 compared to males, but also see significant increases in the apoE3 isoform with age (Nielsen et al., 2017). In the same study, higher ratios of apoE4/apoE3 were negatively associated with CMRglc and GMV. These results may point toward an important role for peripheral apoE levels in modulating brain health and may offer insight into the higher risk of AD in women, particularly women with E4 (Altmann et al., 2014; Nielsen et al., 2017).

Several recent studies have explored the connection between E4, glucose metabolism and amyloid pathology. For example, by comparing FDG-PET data using β-amyloid as a continuous variable, Carbonell et al. (2016) showed that E4 and β-amyloid have a strong association with glucose hypometabolism during early AD stages. Cognitively normal E4 carriers have increased Aβ deposition, with a nonlinear relationship with age; albeit subtle effects on GMV and glucose metabolism compared to FDG-PET scans of other cognitively normal noncarriers (Gonneaud et al., 2016).

Other groups have recently begun to probe the relationship of peripheral glucose regulation and insulin sensitivity to cerebral metabolism. For example, Foley et al. (2016) showed that in E4 carriers, the degree of glucose dysregulation (measured by fasting blood glucose concentration and HbA1C) correlates with reduced cortical thickness; in fact, those diagnosed with diabetes demonstrated a level of cortical thinning comparable to that of preclinical AD. Additionally, impaired glycemia (defined here as a fasting glucose ≥100 mg/dL) and E4 genotype are independent risk factors for cerebral amyloid deposition in cortical regions, but do not appear to have an additive effect (Morris et al., 2016). E4 also confers a greater risk of age-related white matter hyperintensities in diabetics aged 73–76 years and acts as a predictor for progression of white matter hyperintensities (Cox et al., 2017). Finally, post-mortem studies of young adult E4 carriers showed upregulation of several transporters (GLUT1, GLUT3 and MCT2), metabolic enzymes (hexokinase, SCOT and AACS), and mitochondrial complexes I, II, IV, suggestive of inherent apoE-associated alterations in metabolism (Valla et al., 2010; Perkins et al., 2016).

Increasing evidence suggests that cognitive impairment resulting from IR and E4 share common neuropathological features and involve similar changes in metabolism and cerebrovascular function. For instance, the cerebrovascular pathology observed in diabetic patients and in individuals with E4 show significant overlap (Walker and Harrison, 2015) and both IR and E4 have been independently associated with brain glucose hypometabolism and reduced cerebral blood flow (CBF; Thambisetty et al., 2010; Filippini et al., 2011; Pallas and Larson, 1996; Chung et al., 2015). Additionally, these two risk factors appear to interact to impair cognition and drive neurodegeneration (Peila et al., 2002; Dore et al., 2009; Salameh et al., 2016; Johnson et al., 2017a,b). Along these lines, a number of recent studies directly implicate E4 in pathways of insulin signaling (Wolf et al., 2013). For example, in both human apoE mice and postmortem human brain tissue, E4 reduced the expression of insulin signaling proteins such as IRS1 and Akt (Ong et al., 2014; Keeney et al., 2015). In a mouse model of AD, E4 expression accelerated cognitive deficits and exaggerated impairments in insulin signaling (Chan et al., 2015, 2016). Importantly, a recent study by Zhao et al. (2017) showed that E4 directly impairs cerebral insulin signaling in an age-dependent manner, and that peripheral IR and E4 also acted synergistically to impair insulin signaling in the brain. Finally, E4+ individuals do not cognitively benefit from intranasal insulin administration, suggestive of brain IR (Reger et al., 2006; Hanson et al., 2015b). Together, these findings may suggest a metabolic dysregulation in early life stages preceding disease onset (Perkins et al., 2016).

## *APOE* and Brain Lipid Metabolism

ApoE serves as the primary lipid carrier protein in the brain, carrying cholesterol synthesized from astrocytes to neurons in HDL-like lipoprotein particles. These lipid carrying lipoproteins have been shown to interact with Aβ (Sanan et al., 1994). In both transgenic AD mouse models and in post-mortem AD tissue, apoE and its corresponding cholesterol were shown to co-localize with Aβ plaques (Panchal et al., 2010; Lazar et al., 2013). Interestingly, lipid associated E4 has a higher Aβ binding affinity than the delipidated isoforms (Sanan et al., 1994). While this may suggest that cholesterol is involved in E4 driven AD pathology, the mechanism by which apoE-shuttled cholesterol interacts with Aβ is unclear.

In addition to *APOE* associated alterations in brain cholesterol, studies have shown that there is an isoform-dependent usage of fatty acids in both mice and humans. Arbones-Mainar et al. (2016) showed that E4 mice exhibit a metabolic shift toward fatty acid oxidation compared to controls using indirect calorimetry. In humans, it has been reported that E4 individuals β-oxidize uniformly labeled docosahexaenoic acid at higher rates than age- and disease-matched controls (Chouinard-Watkins et al., 2013). E4 expressing mice have also shown to have dysregulated fatty acid synthesis in the entorhinal cortex (Nuriel et al., 2017). Specifically, targeted metabolomics of E4 entorhinal cortices revealed significant changes in multiple glycerolipid and glycerophospholipid species, (Johnson et al., 2017a) as well as seven fatty acid species, (Nuriel et al., 2017) when compared to E3 mice. Thus, in addition to the glucose and insulin impairments described in the previous section, these studies point to a potential lipid mismanagement in E4 carriers which may contribute to AD pathogenesis.

## *APOE*, Cerebral Blood Flow and Cerebral Amyloid Angiopathy

Similar to dynamic vasculature meeting metabolic needs through hyperemic action in skeletal muscle, a related theory has been posited in the brain as so called “neurovascular coupling”. These events are the observed increases in CBF to meet hyperactive neuronal activity. Initially thought to be a response to an oxygen deficit, there is now evidence suggesting direct action of various vasoactive agents on the local vasculature to modulate CBF. Neurovascular coupling is the basis for routinely used imaging platforms including functional magnetic resonance imaging (fMRI). The blood oxygen level dependent (BOLD) contrast response represents an fMRI signal comprised of both a blood flow and metabolic component, as each voxel reflects changes in deoxyhemoglobin and CBF.

Some studies suggest that cerebral hypoperfusion precedes, and possibly contributes to, the onset of dementia (Ruitenberg et al., 2005). Because metabolic rate and CBF are coupled, alterations in cerebral metabolism are likely to affect CBF (Koehler et al., 2009). However, it remains unclear whether cerebral hypoperfusion is a driver of cognitive decline, or whether the deficits simply reflect diminished metabolic demand due to aging and/or neurodegeneration. Given the importance of an efficient and responsive vascular system, it has been proposed that the accelerated AD pathogenesis associated with E4 may result from detrimental cerebrovasculature effects (Tai et al., 2016).

It is well established that CBF is decreased in AD patients (Celsis et al., 1997; Roher et al., 2012). However, both increased (Scarmeas et al., 2005; Thambisetty et al., 2010; Filippini et al., 2011) and decreased (Wierenga et al., 2013; Zlatar et al., 2014) CBF has been observed in individuals with E4, with results differing depending on age (Filippini et al., 2011). Reduced CBF in multiple brain regions has been observed in elderly E4 carriers relative to non-carriers (Filippini et al., 2011). Cognitively normal E4 individuals also show sharper age-related declines in regional CBF (Thambisetty et al., 2010) and *APOE* modifies the association between cognitive function and age-related changes in CBF (Wierenga et al., 2013). In addition to these differences in resting CBF, several studies have shown differences in functional activation, as measured by BOLD fMRI, in middle aged and older E4 individuals (Scarmeas et al., 2005). Functional differences in CBF have been noted in individuals as early as their 20s (Scarmeas et al., 2005) suggesting that E4-associated alterations in brain physiology occur early in life—in the absence of gross neuropathological changes and preceding cognitive impairments (Di Battista et al., 2016).

Evidence from studies using human apoE mice also highlight CBF as a potential link between E4 and impaired cognition. For example, Wiesmann et al. (2016) recently used a flow-sensitive MRI technique to show that 18-month old E4 mice have reduced CBF compared to WT mice. Using MRI, Lin et al. (2017) similarly showed that compared to WT mice, E4 mice have reduced CBF. Furthermore, they showed improvements in CBF in E4 mice following treatment with rapamycin, a pleiotropic compound with various metabolic effects, and provided evidence that the blood brain barrier (BBB) is involved in mediating these effects (Lin et al., 2017). Our own group recently showed that both diet-induced IR and E4 decreased cerebral blood volume (CBV) as measured with optical microangiography (Johnson et al., 2017b). We further demonstrated that an oral glucose gavage selectively improved cognitive performance in E4 mice with IR, and that this spike in blood glucose resulted in a significant increase in CBV. Interestingly, our results in this mouse model of human apoE mirrored a recent clinical research study, in which E4 carriers showed acute cognitive benefits from a high glycemic index meal (Hanson et al., 2015a). However, it should be noted that not all studies of CBF or CBV in human apoE mice have been in consensus. For example, a recent study using a steady-state gadolinium-enhanced fMRI technique showed that, compared to E3 mice, aged E4 mice were found to have higher CBV in the hippocampal formations (Nuriel et al., 2017).

BBB dysfunction can lead to impairments in microvascular function, and thus represents a potential pathway leading to neurodegeneration and AD (Zlokovic, 2013). In fact, multiple studies have linked *APOE* genotype with BBB function, with E4 leading to higher BBB permeability, decreased cerebral vascularization, thinner vessel walls and reduced CBF (Bell et al., 2012; Alata et al., 2015). Importantly, these E4-associated vascular defects were observed as early as 2 weeks of age (Bell et al., 2012) well preceding the neuronal and synaptic dysfunction that is observed in these mice in late age.

Apart from its effect on AD risk, E4 has also been independently linked to the development of cerebral amyloid angiopathy (CAA). A majority of AD brains show CAA, which is a result of amyloid deposition within the walls of small vessels in the leptomeninges and brain parenchyma. A recent study from Nielsen et al. (2017) showed increased incidence of CAA in ApoE4 postmortem brain tissues. Interestingly, they also found an association with E2, but this finding is not consistent across other studies (Rannikmae et al., 2014). CAA has been studied in the context of AD mouse models as well. Deletion of murine *APOE* in two mouse models of Aβ deposition resulted in abolishment of Aβ deposits in the brain parenchyma and cerebrovasculature (Holtzman et al., 2000). This deletion also resulted in less CAA-associated microhemorrhage (Fryer et al., 2005). These data demonstrate that apoE facilitates the formation of cerebrovascular plaques, which are pathological hallmarks of CAA. As compliance decreases with increased deposition of insoluble material, it is probable that cerebrovascular amyloid has substantial effects on the hemodynamics of the brain microvasculature. Could deposition of Aβ in the microvascular walls be a primary cause of the decreased CBF in E4 individuals? Questions such as these highlight the need for further studies examining CBF in the context of CAA.

## Limitations of Cerebral Metabolic Imaging, Alternative “Imaging” Approaches and Future Directions

While they have provided an invaluable knowledge base, each brain imaging technology described above comes with its own set of limitations. Of particular importance in regards to the E4-associated phenomenon of cerebral glucose hypometabolism, are the limitations to biological interpretation of FDG-PET measures. Mainly that CMRglc as determined by FDG-PET is based on blood flow, 2-deoxyglucose (not glucose) transport out of the bloodstream, and phosphorylation of 2DG by the enzyme hexokinase. Thus, the process and its interpretation are restricted to the initial biochemical steps of glycolysis. In theory, the net rate of 2DG uptake is equal to the net rate of the entire glycolytic pathway at steady state (Reivich et al., 1969; Sokoloff et al., 1977; Sokoloff, 1977, 1984; Phelps et al., 1979). However, limited resolution means the cell type(s) responsible remain unknown, and there is no information on whether glucose is eventually converted to ATP in mitochondria, enters the pentose phosphate pathway, is stored as glycogen, or converted to lactate (Mosconi, 2013). Thus, future studies aimed at tracing glucose and other metabolites to their eventual fate will be critical in expanding our understanding of *APOE*’s effects on cerebral metabolism.

Outside of traditional PET- and MRI-based approaches, a few other imaging modalities have been applied to the question of *APOE* influences on cerebral metabolism. For example, magnetic resonance spectroscopy (MRS) has been used by several groups to examine common metabolite concentrations in control and AD individuals of various *APOE* genotypes, as well as human apoE mice. Results have been conflicting, with some groups showing increased choline/creatine and myo-inositol/creatine ratios in E4 carriers (Gomar et al., 2014; Riese et al., 2015) while others showed no *APOE* differences in measured metabolites (Kantarci et al., 2000; Suri et al., 2017). Finally, Dumanis et al. (2013) used MRS in human apoE mice to show a decrease in production of glutamate and increased levels of glutamine in E4 mice.

New applications of established imaging modalities are also providing novel insight into cerebral metabolism. For example, a recent study by Shokouhi et al. (2017) utilized a novel FDG-PET analysis, the regional FDG time correlation coefficient (rFTC) to sensitively measure longitudinal changes in metabolism in cognitively normal individuals. By capturing within-subject similarities between baseline and follow-up regional radiotracer distributions, they showed that rFTC decline was significantly steeper in E4 carriers compared to noncarriers (Shokouhi et al., 2017). PET can provide measurement of not only CMRglc, but also metabolic rate of oxygen (CMRO_2_), thereby allowing estimation of glucose metabolism outside of oxidative phosphorylation, or aerobic glycolysis. A series of articles by Raichle and colleagues have shed new light on the importance of cerebral rates of aerobic glycolysis by defining a new measure of aerobic glycolysis, the glycolytic index (GI), and applying this measure to pertinent studies of regional metabolic variability, amyloid deposition, and cognitive activation. Vaishnavi et al. (2010) showed strong regional variations in aerobic glycolysis, with two cortical regions (the default mode network and areas in the frontal and parietal cortex), showing the highest rate. Further, the areas of the normal brain that demonstrate the highest rates of aerobic glycolysis show near complete overlap with areas of the AD brain that preferentially accumulate amyloid, and it has thus been suggested that impairments in aerobic glycolysis may contribute to AD pathophysiology (Vlassenko et al., 2010). Finally, Shannon et al. (2016) combined fMRI and PET to examine the metabolic profile of activated brain areas before and after a task, and demonstrate that aerobic glycolysis is indeed enhanced in areas undergoing learning induced plasticity. Interestingly, declines in cerebral glucose utilization are greater than those in blood flow and oxygen consumption in the early stages of AD (Lying-Tunell et al., 1981; Hoyer et al., 1991; Fukuyama et al., 1994; Blass, 2002). This discrepancy in the initial stages of the disease may suggest that changes in aerobic glycolysis are an influential metabolic feature of early AD, and raises several important questions, including: Do differences in aerobic glycolysis underlie the hypometabolism observed in young E4 carriers?

Finally, other non-imaging methods of “visualizing” metabolic changes may prove instrumental in elucidating *APOE*-driven alterations in cerebral metabolism. For example, two groups have applied metabolomic analyses to brain tissue from human apoE expressing mice (Johnson et al., 2017a; Nuriel et al., 2017). Nuriel et al. (2017) used a mass spectrometry (MS) based metabolomics technique to report an E4-associated downregulation of several fatty acid species and an upregulation of multiple TCA-cycle metabolites, among other changes. Our own recent study applied an integrated ‘omics approach to provide insight into the metabolic pathways altered in E4 brains (Johnson et al., 2017a). Combining measures of DNA hydroxymethylation and MS-based metabolomics, we identified novel E4-associated alterations in multiple pathways, most notably purine metabolism, glutamate metabolism, and the pentose phosphate pathway (Johnson et al., 2017a).

## Summary

Over the years, the neuropathological characterization of AD has expanded beyond the classic descriptions of amyloid and tau pathology to include metabolic and vascular dysfunction. Advances in brain imaging, most notably PET and MRI, have been invaluable in broadening our understanding of AD pathophysiology. Specifically, the metabolic and cerebrovascular dysfunction described by these studies includes reductions in brain glucose uptake, blunted insulin signaling, alterations in brain lipid metabolism, loss of BBB integrity and deficits in CBF. As reviewed above, many similar alterations have been observed in E4+ individuals, sometimes early in life and often even in the absence of cognitive impairment. Perhaps reflected in the sum of these findings is an inherent inability of E4+ individuals to efficiently regulate cerebral metabolism; although whether it occurs at the level of the BBB or cellular uptake, oxidative phosphorylation, aerobic glycolysis or elsewhere remains unclear. Future studies aimed at expanding the important knowledge base established by PET, MRI and other traditional imaging techniques will be critical in better understanding how E4 drives metabolic dysfunction in AD, and essential in identifying new therapeutic targets to correct these deficiencies in order to delay or prevent AD.

## Author Contributions

JB, BF, HW and LJ wrote and edited the manuscript. All authors read and approved the final version of the manuscript.

## Conflict of Interest Statement

The authors declare that the research was conducted in the absence of any commercial or financial relationships that could be construed as a potential conflict of interest.
